# Detecting Subtle Osborn Waves in Hypothermia: A Case Report

**DOI:** 10.7759/cureus.67090

**Published:** 2024-08-17

**Authors:** Ashot Batikyan, Shiny Teja Kolli, Minas Sakellakis, Aleksan Khachatryan, Hakob Harutyunyan, Vahagn Tamazyan

**Affiliations:** 1 Department of Internal Medicine, Albert Einstein College of Medicine, Jacobi Medical Center/North Central Bronx Hospital, New York, USA; 2 Department of Cardiology, Icahn School of Medicine at Mount Sinai/Mount Sinai Hospital, New York, USA; 3 Department of Internal Medicine, Maimonides Medical Center, New York, USA

**Keywords:** abnormal ekg, clinical cardiology, hypothermia, osborn waves, j waves

## Abstract

J waves, or Osborn waves, are a notable EKG finding in hypothermia, often appearing as prominent deflections but sometimes manifesting subtly. We report a 78-year-old female with moderate hypothermia (87.9°F) presenting with sinus bradycardia and subtle J waves on her EKG. After rewarming, these J waves resolved. Hypothermia management should prioritize gentle handling to avoid arrhythmias and ensure rapid rewarming during cardiopulmonary resuscitation. Continuous monitoring is crucial, as J waves can indicate a higher risk of ventricular fibrillation and cardiac arrest.

## Introduction

The J wave is a well-known EKG finding in hypothermia. It usually appears as a prominent deflection between the end of the QRS complex and the beginning of the ST segment on the EKG [1], but on rare occasions, it can manifest as subtle changes that are frequently overlooked. While J waves are not specific for hypothermia [2], their presence may be correlated with a higher risk of ventricular fibrillation and cardiac arrest [3]. In this report, we present a case of moderate accidental hypothermia in a 78-year-old female highlighting valuable diagnostic and prognostic aspects of J waves in hypothermia.

## Case presentation

A 78-year-old female patient with a past medical history of hypertension, hyperlipidemia, uncontrolled type 2 diabetes mellitus, vascular dementia, and schizophrenia was brought to the emergency department after being found confused sitting near the open window for several hours. On arrival, her rectal temperature was 87.9°F. She was bradycardic with a heart rate of 53 beats per minute, blood pressure of 150/84, respiratory rate of 16 breaths per minute, and oxygen saturation of 96% on room air. On physical examination, she was alert and only oriented to herself, whereas at baseline, she was oriented to person and place, but not time. The remainder of the physical examination was unremarkable. Laboratory tests on presentation, summarized in Table 1, revealed several abnormalities, including mild hyperkalemia, mild hyperglycemia, mixed respiratory acidosis with metabolic acidosis, and mild transaminitis. All other labs, including high-sensitivity troponin, were within normal limits. A chest X-ray was unremarkable.

**Table 1:** Abnormal laboratory results on presentation. VBG: Venous blood gas; ALT: alanine transaminase; AST: aspartate aminotransferase; ALP: alkaline phosphatase

Laboratory parameter	Abnormal result	Reference range
Potassium	5.5 mEq/L	3.5 - 5.0 mEq/L
Glucose	227 mg/dL	70 - 105 mg/dL
Hemoglobin A1C	13.6 %	3.9 - 6.9 %
Bicarbonate	22.4 mEq/L	24 - 30 mEq/L
PCO2 in VBG	50 mmHg	38 - 41 mmHg
pH in VBG	7.27	7.32 - 7.43
ALT	85 U/L	1 - 40 U/L
AST	63 U/L	1 - 40 U/L
ALP	170 U/L	35 - 104 U/L

**Figure 1:**
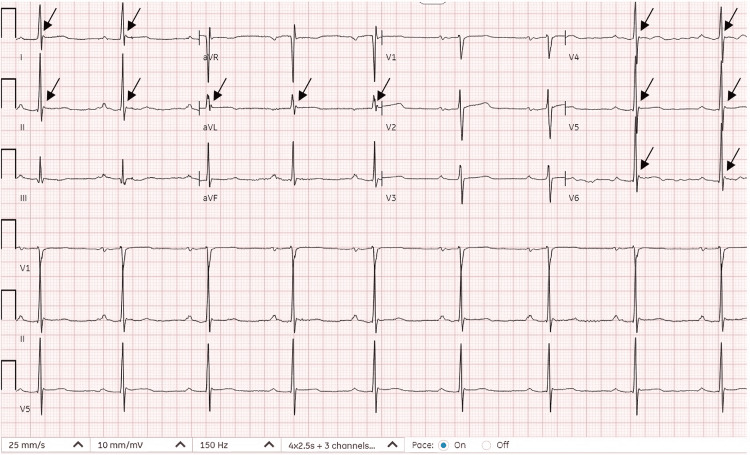
Sinus bradycardia with a heart rate of 52 beats per minute and first-degree atrioventricular block. Black arrows depicting subtle J waves visible in several leads being most prominent in lateral leads.

The EKG upon presentation is shown in Figure 1.

**Figure 2:**
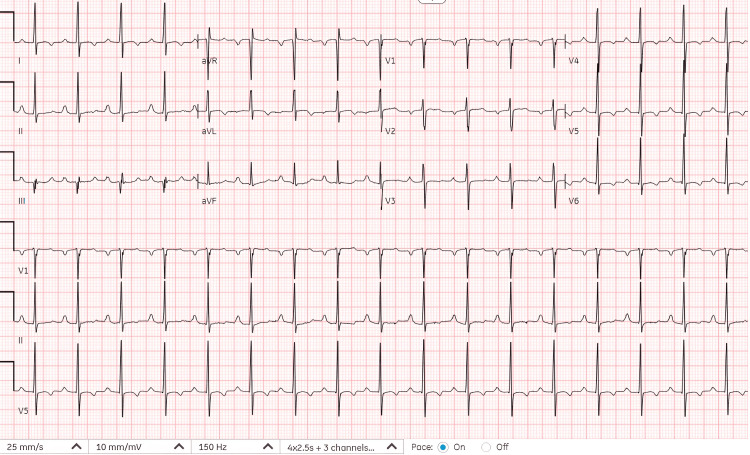
Sinus tachycardia with a heart rate of 105 beats per minute and non-specific T wave changes in lateral leads consistent with her baseline EKG. With normothermia, J waves disappeared and first-degree atrioventricular block resolved.

The patient was transferred to the intensive care unit and external rewarming with a warming blanket was initiated. She was placed on telemetry. An infectious workup, including urinalysis and urine and blood cultures, returned negative. After rewarming to 97.9°F, a repeat EKG was performed, as shown in Figure 2, which was consistent with her previous EKGs from prior hospitalizations. Repeat laboratory studies, including high-sensitivity troponin, were within normal limits. No arrhythmias were observed on the telemetry review. Her mental status returned to baseline with rewarming.

## Discussion

Hypothermia is defined as a core temperature of less than 95°F. It has been classified as mild (90-95°F), moderate (82-90°F), and severe (<82°F). According to this classification, on admission, the patient had moderate hypothermia [1].

Apart from arrhythmogenic complications caused by hypothermia, hypothermia itself can cause a variety of EKG alterations such as the prolongation of RR, PR, QRS, and QT intervals due to slowed impulse conduction through potassium channels [2], as well as the appearance of J waves. From the abovementioned manifestations, the patient had sinus bradycardia, first-degree atrioventricular block, and J waves. The J-wave, or Osborn wave, is a hypothermia-associated deflection that appears between the end of the QRS complex and the beginning of the ST segment [1]. The reported incidence of Osborn waves in cases of hypothermia below 86°F is up to 80% [3]. The J wave is mostly prominent in inferior and precordial leads and is roughly proportional to the degree of hypothermia [4,5]. The appearance of the J wave on EKG is thought to result from a prominent action potential notch in the epicardium mediated by the transient outward current (Ito) [5]. It has been suggested that the presence of J waves might be correlated with a higher incidence of ventricular fibrillation and cardiac arrest [6].

Several other conditions such as hypercalcemia, brain injury, subarachnoid hemorrhage, damage to sympathetic nerves in the neck, and cardiopulmonary arrest from oversedation have also been reported to cause J waves on EKG [7].

Hypothermia management varies from passive external rewarming to active invasive internal rewarming techniques including ECMO depending on the degree of hypothermia and the patient’s response. Rough handling of patients, especially moderate or severe hypothermic ones, can precipitate arrhythmias, including ventricular fibrillation, that are often unresponsive to defibrillation and medications. Cardiopulmonary resuscitation should start without medications and with only one defibrillation attempt until the patient is rewarmed to 86°F. Once this temperature is reached, advanced cardiopulmonary resuscitation with defibrillation and medications can be resumed [8]. 

The relationship between hypothermia and mental health issues is particularly relevant in patients with cardiovascular conditions. Cognitive impairments such as dementia, as seen in this patient, can increase the risk of hypothermia due to decreased awareness and impaired ability to seek warmth or appropriate care. Additionally, psychiatric conditions like schizophrenia may further complicate the management and prevention of hypothermia in such patients and lead to poor cardiovascular outcomes [9,10]. Continuous monitoring and a holistic approach to managing these comorbidities are crucial for optimal outcomes.

## Conclusions

J waves are suggestive but not pathognomonic for hypothermia. Occasionally, they can appear as subtle changes in the EKG, and physicians should be aware of this type of EKG manifestation of hypothermia given its predictive potential for malignant arrhythmias. Continuous monitoring of EKG and electrolytes while treating hypothermia is required to ensure the optimal clinical outcome.
